# Anti‐Glycation and Anti‐Aging Efficacy of Newly Synthesized Antioxidant With Autophagy Stimulating Activity

**DOI:** 10.1111/jocd.70240

**Published:** 2025-05-19

**Authors:** Kayoung Shin, Yeonjae Kim, Sungwoo Kim, Hyun Jung Kim, Sekyoo Jeong, Mi Jang, Jeong Ho Park, Gaewon Nam

**Affiliations:** ^1^ Research Team Incospharm Corp Daejeon South Korea; ^2^ Department of Dermatology Chungnam National University Sejong Hospital Sejong South Korea; ^3^ Department of Chemical & Biological Engineering Hanbat National University Daejeon South Korea; ^4^ Bio‐Living Engineering Major Global Leaders College, Yonsei University Seoul Republic of Korea

**Keywords:** advanced glycation endproducts (AGEs), antioxidant, autophagy, clinical efficacy testing, cosmetic dermatology, N‐dichloroacetyl tryptamine (NdCAT)

## Abstract

**Background:**

The accumulation of advanced glycation end products (AGEs) in aged skin and their pro‐aging effects suggest the potential application of anti‐glycation ingredients as skin anti‐aging agents.

**Aims:**

This study evaluated the anti‐aging efficacy of a newly developed anti‐glycation ingredient with antioxidant and autophagy‐stimulating activities through in vitro, ex vivo, and clinical efficacy tests.

**Methods:**

AGEs formation in both the cell‐free BSA/glyoxal system and glucose/glyoxal‐treated human epidermal keratinocytes was measured, while the degradation of pre‐formed BSA/AGEs by keratinocytes was assessed. Anti‐glycation and anti‐inflammatory effects were further examined using *an* ex vivo human skin explant model. Clinical anti‐aging effects were analyzed by assessing skin AGE levels, melanin and erythema indices, skin elasticity, and skin hydration levels.

**Results:**

The tested ingredient inhibited AGE formation and accelerated the degradation of pre‐formed AGEs in vitro. A significant reduction in skin AGE levels, reduction of skin melanin and erythema indices, and improvement of skin elasticity and hydration in healthy volunteers were observed after 2 and 4 weeks of test product application.

**Conclusion:**

A newly synthesized antioxidant with autophagy‐stimulating activity exhibited significant anti‐glycation efficacy in both in vitro and clinical studies, suggesting its potential as an effective skin anti‐aging ingredient.

## 1 Introduction

Advanced glycation end‐products (AGEs) are generated through an irreversible cross‐linking of amino groups in proteins, lipids, or nucleic acids with the carbonyl groups of reducing sugars through Maillard reactions. In addition to intrinsic factors, such as chronological aging, extrinsic factors, including exposure to ultraviolet rays [1], pollution, or smoking [2] can promote the generation of AGEs in the skin, ultimately contributing to skin aging. In the dermis, fibrillin‐1 and vimentin have been identified as major glycation targets [3]. Glycation‐induced structural deterioration of these proteins results in dermal stiffening and reduced elasticity [4]. Beyond mechanical damage, AGEs also induce oxidative stress and inflammatory responses, mainly through activation of receptors for AGEs (RAGE) on cell surfaces [5]. AGEs have also been shown to stimulate melanin production in cultured melanocytes and increase pigmentation in an ex vivo human skin model, further emphasizing their pro‐aging effects [6]. Furthermore, elevated levels of AGEs have been repeatedly observed in aged skin [7]. Due to their significant roles in skin aging, the development of anti‐AGE strategies has attracted increasing interest in the cosmetic industry.

Based on the biochemical mechanisms underlying AGE generation, several anti‐AGE strategies have been proposed, such as reducing glycation stressors such as blood sugar levels or high‐sugar food intake [8]. However, in dermatological applications, most studies have focused on identifying natural products with antioxidant activity that can block the oxidative stress‐mediated AGEs. For example, methyl gallate, a methyl ester of gallic acid, and gentiopicroside, an active ingredient from the *Gentianaceae* family, have been reported as potential anti‐glycation compounds [9, 10]. Amino acid derivatives such as L‐Carnosine (beta‐alanine‐histidine) and N‐acetyl‐L‐hydroxyproline have also been shown to have antioxidant and anti‐AGE properties [11, 12]. However, few studies have reported on the specific removal of pre‐formed AGEs.

In addition to other cellular degradation mechanisms such as lysosomal [13], proteosomal [14], and receptor‐mediated pathways [15], the autophagic pathway has also been implicated in AGEs clearance. Autophagy is an evolutionarily conserved, intracellular catabolic process by which dysfunctional organelles, misfolded proteins, and exogenous materials are eliminated via lysosomal degradation. In addition to the well‐known mammalian target of rapamycin (mTOR) inhibitors, such as rapamycin, nutrient deprivation and the activation of cyclic adenosine monophosphate (cAMP) kinases can stimulate autophagic flux. In the skin, various homeostatic responses, including epidermal differentiation, inflammation, immune function, and pigmentation, are regulated by autophagy signaling. A previous study suggested that reduced autophagic activity in aged skin may underlie impaired intracellular degradation of AGEs in the dermis [16]. Recently, it was also reported that stimulation of autophagy activity by either *Nymphaea alba* flower extract or sucrose dilaurate or circular RNA (CirCRNA)‐406 918 can reduce carboxymethyl lysine (CML), a representative AGE, in cultured epidermal keratinocytes [17] and AGEs‐BSA in photoaged dermal fibroblasts as well [18]. These findings suggest that activation of autophagy may not only alleviate the detrimental effects of AGEs but also help to reduce the skin AGEs level by enhancing cellular clearing mechanisms.

Through targeted screening of potential antioxidants, we recently identified a series of tryptamine derivatives as potent antioxidants. Among these, N‐dichloroacetyl tryptamine (NdCAT) exhibited notable antioxidant activity and significant autophagy‐stimulating potential. Based on these dual properties, we hypothesized that NdCAT could serve as a novel anti‐glycation and skin anti‐aging compound—both by preventing AGEs formation through its antioxidant action and by promoting the degradation of pre‐formed AGEs by autophagy stimulation. In this study, we evaluated the anti‐AGE effects of NdCAT using cell‐free and in vitro models, as well as an ex vivo human skin explant model and a clinical study.

## 2 Materials and Methods

### 2.1 Synthesis of N‐Dichroloacetyl Tryptamine (NdCAT)

For the synthesis of N‐dichloroacetyl tryptamine, dichloroacetyl chloride (500 mg, 0.38 mL, 2.95 mmol) and triethylamine (TEA, 4.86 mmol) were added to 5 mL N, N‐dimethylformamide (DMF) in argon gas at room temperature. Tryptamine (520 mg, 3.25 mmol) was added to the solution and stirred at room temperature for 5 h. After adding 50 mL of H_2_O to the reaction solution, organic phase extraction was performed three times using 10 mL of dichloromethane (DCM). Anhydrous MgSO_4_ was added to the separated organic phase and filtered, and the anhydrous organic phase was removed using a rotary evaporator under vacuum conditions. The product was separated and purified by silica gel column chromatography (DCM:methanol = 8:1) (310 mg, yield 67%), and the identity was confirmed by H‐NMR (1H‐NMR (400 MHz, CDCl3): δ 2.99 (t, J = 6.8 Hz, 2H), 3.69 (q, J = 6.8 Hz, 2H), 5.82 (s, 1H), 6.69 (s, 1H), 6.97 (s, 1H), 7.12 (t, J = 8.0 Hz, 1H), 7.20 (t, J = 8.0 Hz, 1H), 7.34 (d, J = 8.0 Hz, 1H), 7.58 (d, J = 8.0 Hz, 1H), 8.27 (s, 1H)).

### 2.2 Materials

Glyoxal, D‐glucose, ascorbic acid, and bovine serum albumin (BSA) were purchased from Sigma‐Aldrich (St. Louis. Mo, USA). OxiSelect AGE ELISA kit was provided by Cell Biolabs Inc. (San Diego, CA. USA). All other reagents and chemicals used were of analytical grade. Normal human epidermal keratinocytes (NHEKs), EpiLife culture medium and Human Keratinocyte Growth Supplement (HKGS) were purchased from Thermo Fisher Scientific (Waltham, MA. USA). NHEKs were maintained in EpiLife culture medium supplemented with HKGS and 1% antibiotic mixture under standard cell culture conditions of 37°C and 5% CO_2_ in a humidified atmosphere.

### 2.3 Measurement of Antioxidant Activity

The antioxidant effect of the tested compound was evaluated using the ABTS (2,20‐azino‐bis 3‐ethylbenzthiazoline‐6‐sulfonic acid) antioxidant assay kit (Zen‐Bio Inc., Research Triangle Park, NC, USA) according to the manufacturer's instructions, with slight modifications. Ascorbic acid was used as a positive control.

### 2.4 Measurement of AGEs

AGEs formation under cell‐free conditions was assessed by measuring glycated BSA using the AGEs ELISA kit. A glycation reaction was performed by adding 10 mM glyoxal to a 10 mg/mL BSA solution, with or without the test sample. The reaction mixture was incubated at 37°C for 4 days, and BSA‐AGE formation was quantified using the ELISA kit, according to the manufacturer's protocol.

For in vitro assays, formation of AGEs was also evaluated in cultured normal human epidermal keratinocytes (NHEKs). Cells were seeded in 6‐well culture plates at a density of 1 × 10^5^ cells/well and incubated for 24 h at 37°C in a 5% CO_2_ incubator. After removing the culture medium, the cells were washed with phosphate‐buffered saline solution, and NdCAT (10 μM) was treated for 24 h. Cells were then exposed to 25 mM glucose and 0.5 mM glyoxal for 2 days. AGE levels in the culture medium were measured using an ELISA kit.

### 2.5 Western Blotting Analysis

NHEKs were seeded in 6‐well plates (1 × 10^5^ cells/well) and incubated for 24 h at 37°C in a 5% CO_2_ incubator. After NdCAT (10 μM) treatment for 24 h, cells were lysed using sample buffer (2× Laemmli) (Elpis Biotech Inc.) and cell lysates were separated by electrophoresis on a 15% Tris–HCl Protein Gel (Bio‐Rad, Hercules, CA. USA). Separated bands were blotted onto polyvinylidene fluoride (PVDF) blotting membranes (Roche Diagnostics GmbH, Basel), probed with appropriate antibodies, and detected using enhanced chemiluminescence (GE Healthcare). The intensity of the bands was quantified using an Alliance Mini HD6 system (UVITEC, Cambridge, England).

### 2.6 Ex Vivo Human Skin Explant Model

NativeSkin human skin explant model was purchased from GeneSkin SAS (Toulouse, France). Anonymized human skin samples used in this study were obtained from a healthy female Caucasian donor aged 33 years (skin type: 2), who underwent an abdominoplasty procedure and provided written informed consent. The donor had no history of allergies or dermatological disorders and had not used corticosteroids. The collection, manufacture, and use of skin models for research purposes were formally authorized by the French Ministry of Research (AC‐2017‐2897, October 12, 2017) and approved by the French Ethics Committee (Comité de Protection des Personnes). This study was conducted in accordance with the principles of the Declaration of Helsinki. Immediately following surgery, skin samples were collected, and subcutaneous adipose tissue was removed from the skin. Then, 11 mm diameter punch biopsies were excised and embedded in a proprietary biological matrix in transwells (Millicell, Sigma‐Aldrich) according to the patented NativeSkin procedure developed by Genoskin. The epidermal surfaces of the skin biopsies were left in contact with air, and the dermal compartment was immersed in the matrix. The skin models were cultured in a proprietary and chemically defined hydrocortisone and serum‐free medium in the presence of 100 μg/mL penicillin and streptomycin in a humidified atmosphere of 5% CO_2_ in an incubator at 37°C. Upon arrival at the laboratory, the skin models were acclimatized for 2 h in 12‐well plates containing 1 mL of maintenance medium (Genoskin SAS) in a humidified incubator at 37°C under 5% CO_2_. After 2 h, fresh maintenance medium was added to the culture wells and maintained until further experiments.

As a glycation inducer, 30 μL of methylglyoxal solution (1 mM) was topically applied on the dermal surface of skin tissue. UV irradiation was performed before sample treatment. After the application of methylglyoxal solution, 30 μL of NdCAT solution (10 ppm) was applied on the dermal surface of the skin tissue, which was then incubated at 37°C in a 5% CO_2_ incubator. All treatments were performed once a day for 3 days, and after 72 h of incubation, tissues were harvested for further histological assessments. Each treatment was performed in duplicate.

### 2.7 Histological Analysis

Skin tissues were fixed in 4% neutral buffered formalin (NBF) overnight at 4°C and dehydrated in a graded ethanol series prior to paraffin embedding. For immunohistochemical staining, 5 mm‐thick tissue sections were deparaffinized and rehydrated before heat‐induced epitope revival treatment in an antigen retrieval solution (ab937, abcam, Cambridge, MA, USA) at pH 6.0 for 10 min at 97°C, followed by cooling for 10 min in a cooling chamber. After blocking non‐specific antibody binding using a blocking reagent (X0909, Dako, Glostrup, Denmark), rabbit anti‐AGE antibody (ab23722, abcam), rabbit anti‐human RAGE antibody (ab216329, abcam), and rabbit anti‐human IL‐6 antibody (ab6672, abcam) diluted in an antibody dilution solution (S3022, Dako) were delivered to the sections and incubated in a humidified chamber overnight at 4°C. Then goat anti‐rabbit immunoglobulin (IgG) H&L (Alexa Fluor 488, ab150077, abcam) was applied to the tissue samples, respectively, and subsequently analyzed under a fluorescence microscope (Eclipse Ni‐U, Intenslight C‐HGFI, DS‐Ri2, Nikon, Tokyo, Japan) at 400× magnification. To observe dermal collagen, Masson's trichrome staining was performed according to a previously reported protocol [19].

### 2.8 Clinical Efficacy Testing

A clinical efficacy study (CRA24‐CT0400) was performed involving 21 Asian female subjects (mean age 44.9 years; standard deviation [SD], 2.82), which was approved by the Institutional Review Board of CRA Korea (21Mar2024). The number of participants was decided based on statistical power and effect size calculation, and a sample of 21 was sufficient to detect statistically significant differences within subjects (pre‐ vs. post‐treatment) using paired statistical tests. All studies complied with the World Medical Association's Declaration of Helsinki (2013) concerning biomedical research involving human subjects. Healthy female volunteers without any skin or systemic diseases were enrolled in this study. Candidates who had received retinoids or LASER therapy within the previous 6 months or who participated in other clinical studies were excluded. All volunteers signed an informed consent form and participated in the study after being explained its purpose and protocol. A total of 21 female volunteers were enrolled and completed the study. All the participants were in their 40s. 57.15% of participants (*n* = 12) were classified as normal weight, while 2 and 6 participants were classified as overweight (*n* = 2) (BMI = 23 ~ 24.9) and obese (*n* = 6) (BMI = higher than 25), respectively. One participant was classified as underweight. None of the participants reported smoking. eleven participants reported less than 1 h of sunlight exposure in a day, and 10 participants reported 1–3 h of sunlight exposure in a day.

During the first visit, participants were asked to complete study‐related medical record questionnaires to confirm the inclusion and exclusion criteria, and each participant provided written informed consent. Before instrumental measurements, participants were asked to rest for at least 30 min in a 40%–60% relative humidity (RH)‐and temperature (20°C–24°C) controlled room. After the baseline measurement, participants were administered to use the test product containing 10 ppm NdCAT (Table 1) on the facial area twice a day for 4 weeks. Skin functions and parameters were measured again after 2 and 4 weeks of product usage. Skin AGEs levels were measured using an AGE scanner (DiagnOptics, Netherlands) based on the fluorescent properties of several AGEs [20]. Melanin and erythema indices were measured using Mexameter MX18 (Courage + Khazaka electronic GmbH, Germany), and skin elasticity was measured as the Ur/Ue ratio (%) using a Cutometer Dual MPA 580 (Courage + Khazaka electronic GmbH, Germany). Skin hydration was measured using Skin‐O‐Mat (Cosmomed Medical Beauty GmbH, Germany). After each measurement, corneocytes were collected using a D‐Squame tape (Clinical and Derm LLC. Dallas, TX) for immunofluorescence staining against AGEs. Before collecting the tape, the skin surface was cleansed with a 70% ethanolic solution, and four tapes were collected from the same site. The first tape was discarded, and the second to fourth tapes were used in further experiments. All measurements were performed in triplicate, and the arithmetic mean was used for statistical analysis.

**TABLE 1:** Formulation information for test product.

Aqua
Butylene glycol
Glycerin
Dipropylene glycol
1.2‐Hexanediol
Propanediol
Polyglyceryl‐10 laurate
Panthenol
Carbomer
Tromethamine
Betaine
Allantoin
Ethylhexylglycerin
Sodium hyaluronate
Hydrolyzed hyaluronic acid
Xanthan gum
Glyceryl acrylate/acrylic acid copolymer
PVM/MA copolymer
Pentylene glycol
Sodium acetylated hyaluronate
Sodium hyaluronate crosspolymer
Hydrolyzed sodium hyaluronate
N‐dichloroacetyl tryptamine (10 ppm)

### 2.9 Immunofluorescence Staining for Corneocytes

Three tapes (second to fourth) were immersed in dissociation buffer (100 mM Tris–HCl (pH 8.0) solution containing 5 mM EDTA, 2% SDS and 20 mM DL‐Dithiothreitol) and left for 15 min at 80°C. After sonication for 10 min at 40°C, the supernatant solution was harvested and centrifuged for 10 min at 5000 × g at ambient temperature. The supernatant solution was discarded, and isolated corneocytes were washed with 1 mL of wash buffer (20 mM Tris–HCl (pH 8.0) solution containing 5 mM EDTA, 2% SDS and 10 mM DL‐Dithiothreitol). After centrifugation for 10 min at 5000 × g at ambient temperature, isolated corneocytes were washed again with PBS and resuspended in 100 μL of PBS. Then, 10 μL of isolated corneocytes solution was applied to the coated slide and dried for 10 min at 45°C; the same procedure was repeated three times. After blocking non‐specific antibody binding using a blocking reagent (X0909, Dako, Glostrup, Denmark) for 15 min at room temperature, rabbit anti‐AGEs antibody (ab23722, abcam) diluted in an antibody dilution solution (S3022, Dako) was added to the slides and incubated in a humidified chamber overnight at 4°C. Then goat anti‐rabbit immunoglobulin (IgG) H&L (Alexa Fluor 488, ab150077, abcam) was applied to the samples and subsequently analyzed under a fluorescence microscope (Eclipse Ni‐U, Intenslight C‐HGFI, DS‐Ri2, Nikon, Tokyo, Japan) at 400× magnification.

### 2.10 Statistical Analysis

Values are expressed as arithmetic mean ± SD. Parametric, two‐tailed, paired Student's t‐tests or nonparametric Wilcoxon signed‐rank tests were performed to compare differences before and after test product usage. The number of participants was determined based on a statistical power calculation to ensure sufficient power to detect significant differences before and after test product usage. All statistical analyses were performed using SPSS Statistics (SPSS v.29.0, IBM SPSS Statistics, USA) with a 95% confidence interval, and *p* values < 0.05 were considered statistically significant.

## 3 Results and Discussion

### 3.1 Prevention of AGE Generation by Antioxidant Activity

The biochemical pathway for the generation of advanced glycation end‐products (AGEs) begins with the relatively slow Maillard reaction between reducing sugars and proteins and/or lipids. Through a Schiff base and/or more stable Amadori products, the resulting reactive carbonyl compounds lead to the formation of AGEs [21]. Additional biochemical processes contributing to AGEs generation include lipid peroxidation and the glycolysis pathway, which generate malondialdehyde or methylglyoxal, respectively. Glycemic, dicarbonyl, and oxidative stresses are the key drivers of AGEs formation, supporting the widespread use of antioxidants to prevent their generation [22, 23].

**FIGURE 1:**
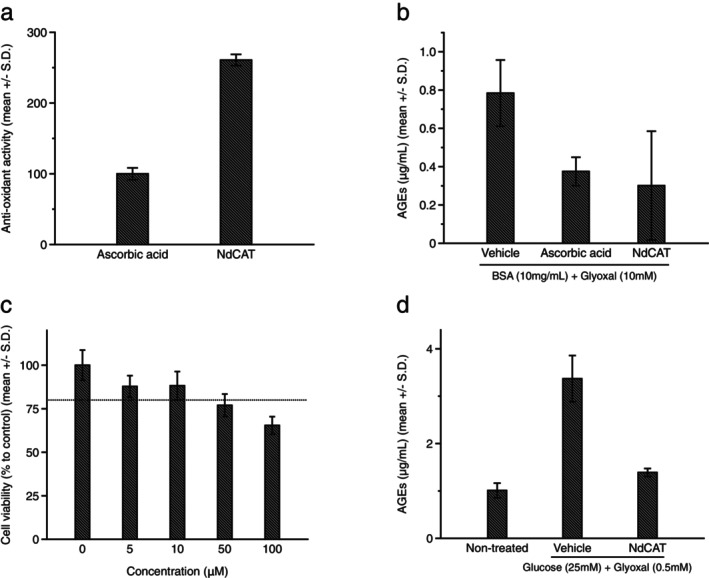
Inhibition of AGEs formation by N‐dichloroacetyl tryptamine (NdCAT). NdCAT demonstrated significant antioxidant activity in ABTS assay (a) and inhibited AGEs‐BSA formation (b). No cytotoxicity was observed at concentrations up to 10 μM (c), and NdCAT prevented cellular AGEs formation in cultured human epidermal keratinocytes (d).

Tryptamine is a core structural component of melatonin (*N*‐acetyl‐5‐methoxytryptamine), which exhibits diverse biological functions, including antioxidant and autophagy stimulating activity [24]. Based on its structural similarity to melatonin, we synthesized a series of tryptamine derivatives and screened them for potential antioxidants for cosmetic applications. Among these, N‐dichloroacetyl tryptamine (NdCAT) was identified as a potent antioxidant. Its activity was evaluated using the ABTS (2,20‐azino‐bis 3‐ethylbenzthiazoline‐6‐sulfonic acid) assay, where NdCAT showed approximately 2.6‐fold higher antioxidant activity compared to ascorbic acid on a molar basis (Figure 1a). Given its significant antioxidant activity, we evaluated the anti‐glycation potential of NdCAT in cell‐free and in vitro cultured human epidermal keratinocyte models. First, cell‐free BSA (bovine serum albumin)‐AGE formation was induced by treating BSA with glyoxal. Both NdCAT (10 μM) and ascorbic acid (1 mM), as a positive control, significantly inhibited BSA‐AGE formation (Figure 1b). In previous studies evaluating the preventing effects of antioxidants on AGEs formation, methyl ester of gallic acid [9], gentiopicroside [10], and N‐acetyl‐L‐hydroxyproline [11] showed similar effects in a cell‐free reaction system. To investigate the effects of NdCAT in cultured human epidermal keratinocytes, we first assessed the cytotoxicity of NdCAT. No cytotoxicity was observed at concentrations up to 10 μM, and this concentration was used for the further experiment (Figure 1c). Co‐treatment with glucose and glyoxal increased the cellular AGEs level approximately 4‐fold higher, and co‐treatment of NdCAT significantly prevented AGEs generation (Figure 1d). These findings demonstrate that the newly synthesized tryptamine derivative, NdCAT, exhibits significant anti‐glycation activity in both cell‐free and cultured keratinocytes due to, at least in part, its antioxidant activity.

### 3.2 Accelerated Elimination of BSA‐AGEs by NdCAT in Skin Cells

**FIGURE 2:**
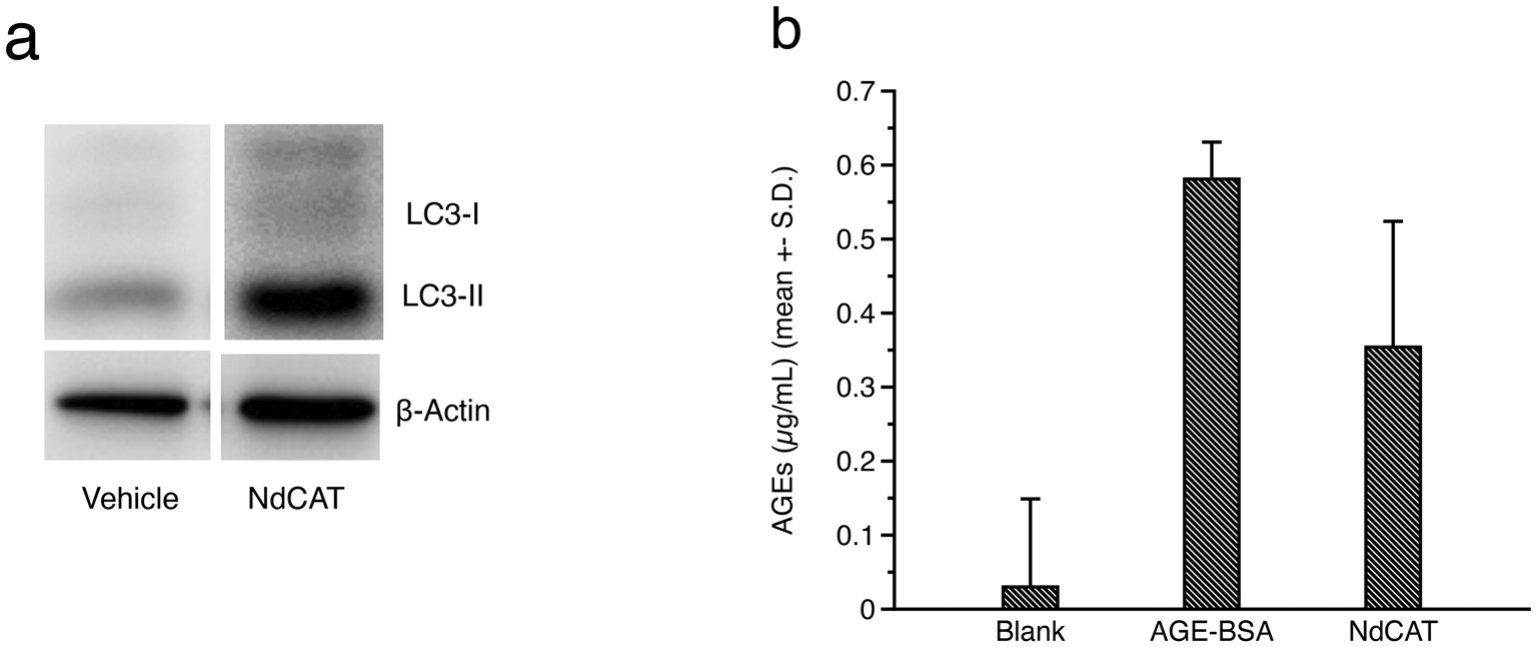
Accelerated degradation of preformed AGEs by autophagy activation. Activation of autophagy signaling, represented by increased amount of LC3‐II, was observed in cultured human epidermal keratinocytes (a). Accelerated degradation of AGEs‐BSA in cultured keratinocytes was also observed in NdCAT‐treated cells (b).

To prevent the cellular accumulation of AGEs within cells, diverse cellular antioxidant systems, including glutathione peroxidases (GPx), glutathione reductase (GR), catalase, peroxiredoxins (Prxs), superoxide dismutase (SODs), along with detoxifying pathways such as the glyoxalase system [25], aldehyde dehydrogenase [26], and aldo‐keto reductase [27], are employed. However, because of the irreversibility of the glycation process, removal of already‐formed AGEs requires proteolytic degradation systems, such as the ubiquitin‐proteasome system (UPS) or autophagy‐mediated lysosomal degradation [28]. Previous studies have reported that treatment with autophagy activators reduces the level of carboxymethyl lysine (CML) in cultured epidermal keratinocytes [17]. Recently, p62‐dependent autophagy has been identified as a conserved pathway for the AGEs clearance [29], suggesting that activation of autophagy can be a new strategy for mitigating AGEs‐related aging symptoms, including those affecting skin. To evaluate the potential AGE‐removal activity of NdCAT, its effects on autophagy signaling in cultured human epidermal keratinocytes were examined by Western blotting and immunohistochemical staining. Measurement of microtubule‐associated protein 1A/1B‐light chain 3 (LC3) conversion from LC3‐I to LC3‐II, which indicates the autophagosomes formation, was increased in cultured keratinocytes by NdCAT treatment, confirming the activation of autophagy signaling (Figure 2a). Immunohistochemical staining for LC3 protein in an ex vivo human skin explant model further confirmed the autophagy activation by NdCAT. Compared to normal tissue, UV‐irradiated skin exhibited reduced LC3 expression in the epidermis. However, topical application of NdCAT restored LC3 expression, especially in the suprabasal layer (Figure 2b). Consistent with previous studies, NdCAT treatment of BSA‐AGEs‐treated keratinocytes significantly reduced AGEs concentrations after 48 h of application (Figure 2c). These results support the anti‐glycation effects of autophagy activators by accelerating the elimination of preformed AGEs in skin cells.

### 3.3 Anti‐AGEs and Anti‐Aging Effects of NdCAT Were Observed in an Ex Vivo Human Skin Explant Model

**FIGURE 3:**
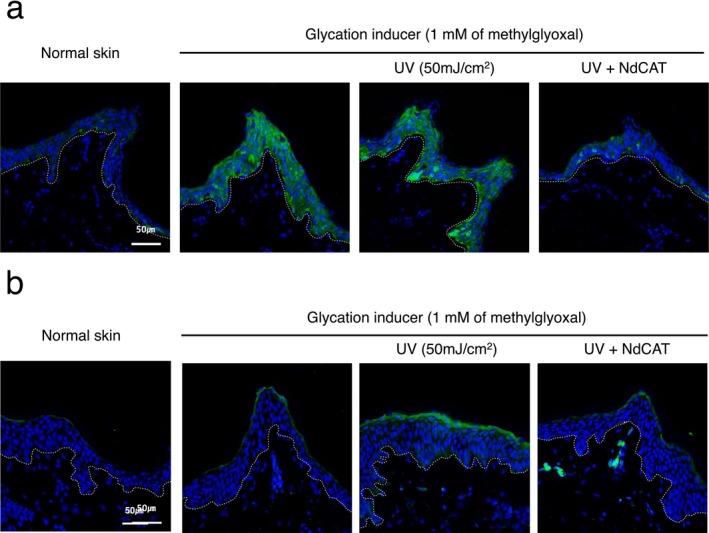
Anti‐glycation activity of NdCAT in ex vivo human skin explant model. Topical application of methylglyoxal, as an experimental glycation inducing agent, increased the formation of AGEs in the epidermis, which was further escalated by UV irradiation. Co‐application of NdCAT significantly reduced AGEs accumulation in the epidermis (a). Similar changes were also observed for the receptor for AGEs (RAGE) in the epidermis (b). (magnification: 400×, dotted line: Dermal‐epidermal junction).

To further investigate the anti‐AGE and potential anti‐aging effects of NdCAT, *an* ex vivo human skin explant model was used. Topical application of methylglyoxal, as a glycation inducer, resulted in an increased expression of AGEs, mostly in the granular layer. Interestingly, additional UV irradiation following glyoxal treatment further intensified AGE accumulation in the basal layer, compared to glyoxal‐only treated tissue. These results suggest that, while chemical glycation stressors can directly induce glycation in the suprabasal layer, UV‐induced oxidative stress can also elicit biological AGEs formation in the basal layer. Consistent with these results, AGEs expression analysis in the UV‐irradiated ex vivo human skin explant model exhibited significantly increased expressions in the basal layer, compared to normal skin [data not shown]. However, topical application of NdCAT prevented AGEs accumulation across all epidermal layers, suggesting that its anti‐glycation activities are mediated by both direct antioxidant activity and activation of the cellular antioxidant system by autophagy signaling activation [30] (Figure 3a).

Immunohistochemical staining showed low expression of RAGE (receptor for AGEs) in the normal epidermis. Upon exposure to glycation stressors, RAGE expression was modestly increased in the upper granular layer, with UV irradiation further increasing its expression. NdCAT treatment suppressed RAGE upregulation as well (Figure 3b). Previously, it was reported that RAGE mediated inflammatory responses by AGEs treatment, and inhibition of RAGE can alleviate the inflammatory responses in epidermal keratinocytes [31].

**FIGURE 4:**
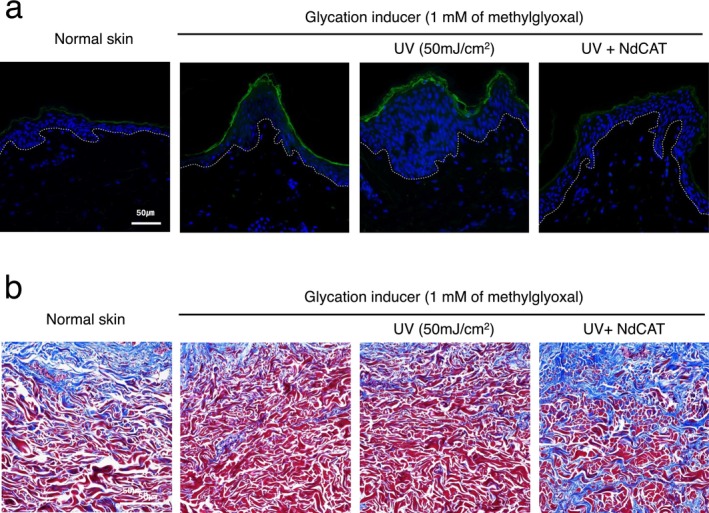
Anti‐aging effects of NdCAT in ex vivo human skin explant model. Immunohistochemical staining against interleukin‐6 (IL‐6) showed increased expression after exposure to glycation inducer and UV, which was significantly attenuated by NdCAT co‐application (a). In Masson's trichrome staining, the decreased expression of dermal collagen in glycation inducer and UV‐irradiated skin tissue was restored by NdCAT application (b). (magnification: 400×, dotted line: Dermal‐epidermal junction).

Deleterious effects of AGEs on dermal extracellular matrix were also reported in a 3D reconstituted human skin model [32]. In this study, we also examined the expression of inflammatory mediators, interleukin (IL‐6), and dermal collagen by immunohistochemical and Masson's trichrome staining. As shown in Figure 4a, the epidermal expression of IL‐6 was significantly increased after glycation stressor and UV treatment. Similar to the AGEs and RAGE expression, topical application of NdCAT markedly prevented the increase in IL‐6, which suggests a potential skin soothing effect of the anti‐glycation ingredient. Masson's trichrome staining also revealed that collagen degradation induced by glycation and UV exposure was mitigated by NdCAT treatment (Figure 4b). Collectively, these results indicate that topical application of NdCAT can provide substantial anti‐aging benefits by preventing AGE accumulation, alleviating inflammatory responses, and protecting dermal collagen deterioration. These data suggest the therapeutic potential of anti‐glycation agents in addressing age‐ and glycation‐related skin changes, including inflammation and wrinkle formation.

### 3.4 Clinical Efficacy Assessment

**FIGURE 5:**
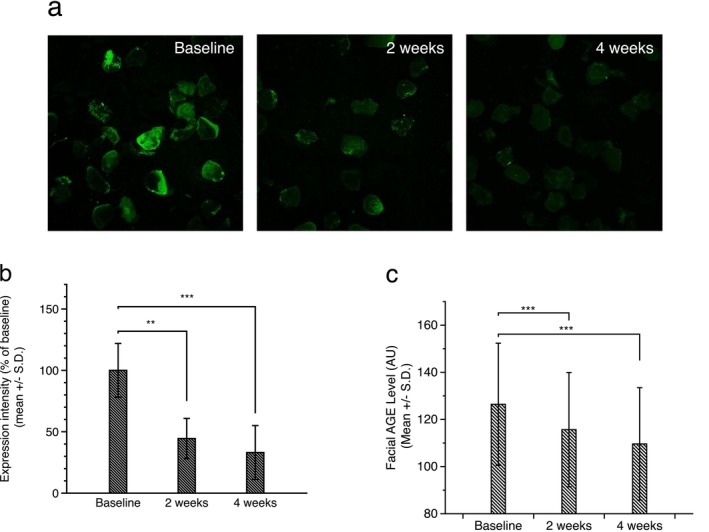
Clinical efficacies of NdCAT on skin AGEs. Immunofluorescence staining against AGEs in corneocytes showed a significant reduction in fluorescence intensity after 2 weeks and 4 weeks of test product application, compared to baseline (a). Image analysis further confirmed the statistical significance (b). AGE scanner measurement also showed a similar reduction in skin AGEs level after 2 weeks and 4 weeks of application (c). (**p < 0.01, ***p < 0.001).

Based on the in vitro and ex vivo results demonstrating the anti‐glycation and anti‐aging potential of NdCAT, a clinical efficacy testing was performed. The primary endpoint of the study was to measure the AGEs levels in the skin. In previous studies, non‐invasive assessments of skin AGEs levels were reported by measuring the skin autofluorescence (SAF), based on the fluorescent properties of several AGEs, such as collagen‐linked fluorescence (CLF), N‐ε‐carboxymethyl‐lysine (CML), N‐ε‐carboxy‐ethyl‐lysine (CEL), and pentosidine [20, 33]. Recently, direct quantification of AGEs in corneocytes isolated by non‐invasive tape‐stripping methods was reported in atopic dermatitis patients [34]; however, to our knowledge, there are no reports measuring AGEs levels in healthy volunteers. Among the 21 volunteers who participated in this study, we randomly selected six participants and measured AGEs levels in corneocytes. Figure 5a shows a representative fluorescence microscopy image of AGEs in isolated corneocytes. Image analysis revealed a significant reduction in fluorescence intensity by 55.5%p at 2 weeks and 66.9%p at 4 weeks after test product application. Consistent with these findings, a significant reduction in the facial SAF value was also observed (Figure 5c). These results suggest that either skin autofluorescence measurement or immunohistochemical staining against AGEs in corneocytes can be used to address skin AGEs levels, and the use of anti‐glycation ingredients can attenuate skin AGEs levels. In this study we successfully established the non‐invasive methods for assessing the skin glycation levels by measuring skin autofluorescence and direct detection of AGEs in isolated corneocytes. This method provides a protocol for evaluating the clinical efficacy of anti‐glycation ingredients or other skin anti‐aging products as well.

**FIGURE 6:**
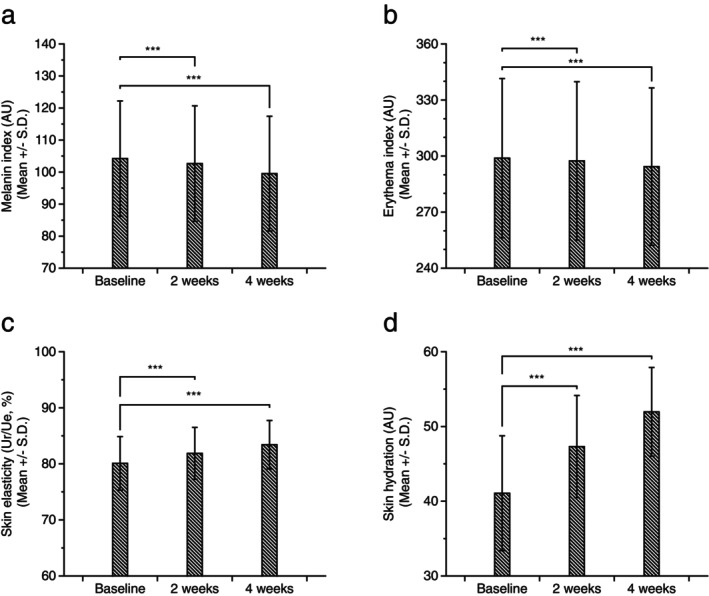
Clinical efficacies of NdCAT on skin aging parameters. Statistically significant reductions of melanin index (a) and erythema index (b) in the facial area were observed after 2‐ and 4‐weeks usage of DCAT. Significant increases in skin elasticity (c) and skin hydration (d) were also observed. (***p < 0.001).

To assess the secondary endpoints of the study, improvements in skin aging‐related parameters, that is, skin elasticity, skin hydration, and skin complexion (melanin and erythema indices), were measured. Changes in skin complexion were evaluated by measuring melanin and erythema indices using Mexameter MX18, which resulted in a significant reduction of both indices at 2 and 4 weeks after treatment (Figure 6a,b). Significant improvements in skin elasticity, evaluated by measuring the Ur/Ue ratio (Figure 6c) [35], and hydration level (Figure 6d) were also observed 2 and 4 weeks after product usage. These results suggest the potential anti‐aging efficacies of the anti‐glycation ingredients. While this study is the first report of clinical evaluation of anti‐AGE ingredient in skin, it has a few limitations. The clinical study was performed over a relatively short period of 4 weeks and a relatively small number of participants were enrolled. Also, only Asian females with certain ages ranging from 40 to 49 were enrolled for the study. Further studies should include larger sample sizes, diverse populations across age groups, genders, and ethnicities to validate the findings.

## 4 Conclusion

Among the various aging stressors in the skin, many endogenous or exogenous factors, such as increased reducing sugar concentration and exposure to UV rays or other oxidative stresses, can stimulate the formation of AGEs either directly or indirectly. Increased AGEs in the skin can further accelerate the skin aging processes by directly interfering with the physiological functions of proteins and/or lipids in the skin. Increased AGE levels also induce inflammatory responses via RAGE signaling. While there are several reports on the potential use of antioxidants for the prevention of AGEs in vitro [36] and ex vivo [32], little has been reported on the clinical efficacy of cosmetic antioxidants on skin AGEs levels. In addition, while there are a few studies suggesting a possible relationship between autophagy signaling and AGEs [37], there are no reports providing any clinical evidence supporting it. In this study, we screened for tryptamine‐based antioxidant molecules and evaluated their ability to stimulate autophagy. As a result, a novel antioxidant with autophagy‐stimulating activity was identified, and its anti‐AGE effects were evaluated in in vitro, ex vivo, and clinical studies. A significant reduction in AGEs formation and accelerated removal of preformed AGEs in cultured human epidermal keratinocytes was observed. A significant reduction in skin AGEs levels, addressed both by immunohistochemical staining and skin autofluorescence measurement, was also observed, which is, according to the authors' search results, the first clinical study data.

Importantly, these results were accompanied by visible clinical improvements in skin aging‐related parameters, suggesting that this antioxidant holds strong potential for commercial application in anti‐aging skincare products. The dual action—anti‐glycation and autophagy stimulation—positions this ingredient as a multifunctional additive that can be integrated into existing formulations such as serums, creams, or ampoules targeted at glycation‐related skin aging. Given that glycation is exacerbated by blood sugar levels and oxidative stressors like UV exposure and smoking, this compound may be particularly valuable for high‐risk consumers, offering brands an opportunity to develop targeted skincare solutions for glycation‐prone or environmentally stressed skin. Further studies are warranted to validate its long‐term efficacy and optimize formulation compatibility for commercial deployment.

## Author Contributions

K.S., Y.K., and S.K. conducted the in vitro and ex vivo studies. H.J.K. and S.J. designed the study and analyzed the results. M.J. and J.H.P. synthesized the tryptamine derivatives and provided the test materials. H.J.K., S.J., and G.N. participated in writing and editing the manuscript. All authors have read and approved the final manuscript.

## Ethics Statement

Authors confirm that the ethical policies of the journal, as noted on the journal's author guidelines page, have been adhered to and the appropriate ethical review committee approval has been received. This study was approved by the Incospharm Corp. All participants followed an appropriately administrated written informed consent process using the IRB‐approved ICF prior to any study procedures, and the study was conducted in compliance with the Declaration of Helsinki and in accordance with all applicable guidelines for the protection of human subjects for research, as outlined in the accepted standards for Good Clinical Practice.

## Conflicts of Interest

The authors declare no conflicts of interest.

## Data Availability

The data that support the findings of this study are available from the corresponding author upon reasonable request.

## References

[1] R. Sultana , A. Parveen , M. C. Kang , S. M. Hong , and S. Y. Kim , “Glyoxal‐Derived Advanced Glycation End Products (GO‐AGEs) With UVB Critically Induce Skin Inflammaging: In Vitro and In Silico Approaches,” Scientific Reports 14, no. 1 (2024): 1843, 10.1038/s41598-024-52037-z. 38246969 PMC10800344

[2] R. P. Van Waateringe , M. J. Mook‐Kanamori , S. N. Slagter , et al., “The Association Between Various Smoking Behaviors, Cotinine Biomarkers and Skin Autofluorescence, a Marker for Advanced Glycation End Product Accumulation,” PLoS One 12, no. 6 (2017): e0179330, 10.1371/journal.pone.0179330. 28632785 PMC5478117

[3] T. Kueper , T. Grune , S. Prahl , et al., “Vimentin Is the Specific Target in Skin Glycation,” Journal of Biological Chemistry 282, no. 32 (2007): 23427–23436, 10.1074/jbc.M701586200. 17567584

[4] T. He , G. J. Fisher , A. J. Kim , and T. Quan , “Age‐Related Changes in Dermal Collagen Physical Properties in Human Skin,” PLoS One 18, no. 12 (2023): e0292791, 10.1371/journal.pone.0292791. 38064445 PMC10707495

[5] M. Iwamura , Y. Yamamoto , Y. Kitayama , et al., “Epidermal Expression of Receptor for Advanced Glycation End Products (RAGE) is Related to Inflammation and Apoptosis in Human Skin,” Experimental Dermatology 25, no. 3 (2016): 235–237, 10.1111/exd.12899. 26566598

[6] E. J. Lee , J. Y. Kim , and S. H. Oh , “Advanced Glycation End Products (AGEs) Promote Melanogenesis Through Receptor for AGEs,” Scientific Reports 6, no. 1 (2016): 27848, 10.1038/srep27848. 27293210 PMC4904211

[7] D. Martinovic , D. Tokic , M. Usljebrka , et al., “The Association Between the Level of Advanced Glycation End Products and Objective Skin Quality Parameters,” Lifestyles 13, no. 2 (2023): 256, 10.3390/life13020256. PMC9961659 36836618

[8] L. Wang , Y. Jiang , and C. Zhao , “The Effects of Advanced Glycation End‐Products on Skin and Potential Anti‐Glycation Strategies,” Experimental Dermatology 33, no. 4 (2024): e15065, 10.1111/exd.15065. 38563644

[9] S. Shin , J. Lee , S. H. Yoon , D. Park , J. S. Hwang , and E. Jung , “Anti‐Glycation Activities of Methyl Gallate In Vitro and in Human Explants,” Journal of Cosmetic Dermatology 21, no. 6 (2022): 2602–2609, 10.1111/jocd.14406. 34418257

[10] C. Chen , X. Liu , L. Li , et al., “Study of the Mechanism by Gentiopicroside Protects Against Skin Fibroblast Glycation Damage via the RAGE Pathway,” Scientific Reports 14, no. 1 (2024): 4685, 10.1038/s41598-024-55525-4. 38409584 PMC10897486

[11] C. Knoblich , K. Dunckelmann , A. Krüger , T. Küper , T. Blatt , and J. M. Weise , “N‐Acetyl‐L‐Hydroxyproline – A Potent Skin Anti‐Ageing Active Preventing Advanced Glycation End‐Product Formation In Vitro and Ex Vivo,” International Journal of Cosmetic Science 46, no. 2 (2024): 297–306, 10.1111/ics.12930. 38013225

[12] C. Girardi , F. Benato , M. Massironi , V. Vindigni , D. Stuhlmann , and M. Massironi , “Evaluation of Human Skin Response to Solar‐Simulated Radiation in an Ex Vivo Model: Effects and Photoprotection of L‐Carnosine,” Photochemistry and Photobiology 100, no. 3 (2024): 733–745, 10.1111/php.13850. 37675862

[13] S. Grimm , L. Ernst , N. Grötzinger , et al., “Cathepsin D Is One of the Major Enzymes Involved in Intracellular Degradation of AGE‐Modified Proteins,” Free Radical Research 44, no. 9 (2010): 1013–1026, 10.3109/10715762.2010.495127. 20560835

[14] G. Aldini , G. Vistoli , M. Stefek , et al., “Molecular Strategies to Prevent, Inhibit, and Degrade Advanced Glycoxidation and Advanced Lipoxidation End Products,” Free Radical Biology and Medicine 47, no. 1 (2013): 93–137, 10.3109/10715762.2013.792926. 23560617

[15] B. Smedsrød , J. Melkko , N. Araki , H. Sano , and S. Horiuchi , “Advanced Glycation End Products Are Eliminated by Scavenger‐Receptor‐Mediated Endocytosis in Hepatic Sinusoidal Kupffer and Endothelial Cells,” Biochemical Journal 322, no. 2 (1997): 567–573. 9065778 10.1042/bj3220567 PMC1218227

[16] Y. Huang , Y. Li , Y. Qu , et al., “UVA‐Induced Photoaging Inhibits Autophagic Degradation by Impairing Lysosomal Function in Dermal Fibroblasts,” Biochemical and Biophysical Research Communications 518, no. 4 (2019): 611–618, 10.1016/j.bbrc.2019.08.103. 31445710

[17] T. Laughlin , Y. Tan , B. Jarrold , et al., “Autophagy Activators Stimulate the Removal of Advanced Glycation End Products in Human Keratinocytes,” Journal of the European Academy of Dermatology and Venereology 34 (2020): 12–18, 10.1111/jdv.16453. 32557807

[18] Y. Qu , M. Wang , J. Lan , et al., “CircRNA‐406918 Enhances the Degradation of Advanced Glycation End Products in Photoaged Human Dermal Fibroblasts via Targeting Cathepsin D,” Photodermatology, Photoimmunology & Photomedicine 39, no. 5 (2023): 487–497, 10.1111/phpp.12887. 37253092

[19] S. Jeong , S. Yoon , S. Kim , et al., “Anti‐Wrinkle Benefits of Peptides Complex Stimulating Skin Basement Membrane Proteins Expression,” International Journal of Molecular Sciences 21, no. 1 (2019): 73, 10.3390/ijms21010073. 31861912 PMC6981886

[20] I. M. Atzeni , S. C. Van De Zande , J. Westra , J. Zwerver , A. J. Smit , and D. J. Mulder , “The AGE Reader: A Non‐Invasive Method to Assess Long‐Term Tissue Damage,” Methods 203 (2022): 533–541, 10.1016/j.ymeth.2021.02.016. 33636313

[21] L. C. de Vos , J. D. Lefrandt , R. P. F. Dullaart , C. J. Zeebregts , and A. J. Smit , “Advanced Glycation End Products: An Emerging Biomarker for Adverse Outcome in Patients With Peripheral Artery Disease,” Atherosclerosis 254 (2016): 291–299, 10.1016/j.atherosclerosis.2016.10.012. 27751506

[22] I. González , M. A. Morales , and A. Rojas , “Polyphenols and AGEs/RAGE Axis. Trends and Challenges,” Food Research International 129 (2020): 108843, 10.1016/j.foodres.2019.108843. 32036875

[23] B. Umbayev , S. Askarova , A. Almabayeva , T. Saliev , A. R. Masoud , and D. Bulanin , “Galactose‐Induced Skin Aging: The Role of Oxidative Stress,” Oxidative Medicine and Cellular Longevity 2020 (2020): 1–15, 10.1155/2020/7145656. PMC7317321 32655772

[24] F. Luo , A. F. Sandhu , W. Rungratanawanich , et al., “Melatonin and Autophagy in Aging‐Related Neurodegenerative Diseases,” International Journal of Molecular Sciences 21, no. 19 (2020): 7174, 10.3390/ijms21197174. 32998479 PMC7584015

[25] G. Aragonès , S. Rowan , S. G. Francisco , et al., “The Glyoxalase System in Age‐Related Diseases: Nutritional Intervention as Anti‐Ageing Strategy,” Cells 10, no. 8 (2021): 1852, 10.3390/cells10081852. 34440621 PMC8393707

[26] Y. Nomi , H. Kudo , K. Miyamoto , et al., “Free Advanced Glycation End Product Distribution in Blood Components and the Effect of Genetic Polymorphisms,” Biochimie 179 (2020): 69–76, 10.1016/j.biochi.2020.09.010. 32946992

[27] S. P. Baba , J. Hellmann , S. Srivastava , and A. Bhatnagar , “Aldose Reductase (AKR1B3) Regulates the Accumulation of Advanced Glycosylation End Products (AGEs) and the Expression of AGE Receptor (RAGE),” Chemico‐Biological Interactions 191 (2011): 357–363, 10.1016/j.cbi.2011.01.024. 21276777 PMC3145413

[28] A. Taylor and E. Bejarano , “Boosting Proteolytic Pathways as a Treatment Against Glycation‐Derived Damage in the Brain?,” Neural Regeneration Research 17, no. 2 (2022): 320–322, 10.4103/1673-5374.317971. 34269200 PMC8463977

[29] G. Aragonès , K. Dasuri , O. Olukorede , et al., “Autophagic Receptor p62 Protects Against Glycation‐Derived Toxicity and Enhances Viability,” Aging Cell 19, no. 11 (2020): e13257, 10.1111/acel.13257. 33146912 PMC7681057

[30] J. Lim , C. J. Lim , S. Kim , et al., “Antiaging and Antioxidant Effects of Topical Autophagy Activator: A Randomized, Placebo‐Controlled, Double‐Blinded Study,” Journal of Cosmetic Dermatology 18, no. 1 (2019): 197–203, 10.1111/jocd.12530. 29524287

[31] C. T. Yang , F. H. Meng , L. Chen , et al., “Inhibition of Methylglyoxal‐Induced AGEs/RAGE Expression Contributes to Dermal Protection by N‐Acetyl‐L‐Cysteine,” Cellular Physiology and Biochemistry 41, no. 2 (2017): 742–754, 10.1159/000458734. 28214842

[32] E. Markiewicz , J. Jerome , T. Mammone , and O. C. Idowu , “Anti‐Glycation and Anti‐Aging Properties of Resveratrol Derivatives in the In‐Vitro 3D Models of Human Skin,” Clinical, Cosmetic and Investigational Dermatology 15 (2022): 911–927, 10.2147/CCID.S364538. 35615726 PMC9126233

[33] R. Meerwaldt , R. Graaff , P. H. N. Oomen , et al., “Simple Non‐Invasive Assessment of Advanced Glycation Endproduct Accumulation,” Diabetologia 47, no. 7 (2004): 1324–1330, 10.1007/s00125-004-1451-2. 15243705

[34] J. Y. Hong , M. J. Kim , J. K. Hong , et al., “In Vivo Quantitative Analysis of Advanced Glycation End Products in Atopic Dermatitis—Possible Culprit for the Comorbidities?,” Experimental Dermatology 29, no. 10 (2020): 1012–1016, 10.1111/exd.14167. 32767581

[35] D. B. Abbas , C. V. Lavin , E. J. Fahy , et al., “Standardizing Dimensionless Cutometer Parameters to Determine In Vivo Elasticity of Human Skin,” Advances in Wound Care 11, no. 6 (2022): 297–310, 10.1089/wound.2021.0082. 34470542 PMC8982109

[36] H. Li , N. Deng , T. Puopolo , et al., “Cannflavins A and B With Anti‐Ferroptosis, Anti‐Glycation, and Antioxidant Activities Protect Human Keratinocytes in a Cell Death Model With Erastin and Reactive Carbonyl Species,” Nutrients 15, no. 21 (2023): 4565, 10.3390/nu15214565. 37960218 PMC10650133

[37] O. Gómez , G. Perini‐Villanueva , A. Yuste , J. A. Rodríguez‐Navarro , E. Poch , and E. Bejarano , “Autophagy and Glycative Stress: A Bittersweet Relationship in Neurodegeneration,” Frontiers in Cell and Development Biology 9 (2021): 790479, 10.3389/fcell.2021.790479. PMC8733682 35004686

